# Bloodletting cupping combined with conventional measures therapy for psoriasis: A systematic review and meta-analysis of randomized controlled trials

**DOI:** 10.3389/fmed.2023.1132928

**Published:** 2023-02-16

**Authors:** Xiaoyu Ma, Dilong Li, Minghui Zhao, Jiaming He, Fan Yang, Jingyan Kong

**Affiliations:** ^1^Department of Basic Theory of Traditional Chinese Medicine, School of Traditional Chinese Medicine, Tianjin University of Traditional Chinese Medicine, Tianjin, China; ^2^Teaching and Research Department of Traditional Chinese Medicine and Cosmetology, School of Traditional Chinese Medicine, Tianjin University of Traditional Chinese Medicine, Tianjin, China

**Keywords:** psoriasis, bloodletting cupping, total effective numbers, PASI, randomized controlled trials, meta-analysis

## Abstract

**Background:**

Psoriasis is an immune-mediated inflammatory disease prone to recurrence. Some studies indicated that bloodletting cupping combined with conventional measures therapy had been proposed as a treatment strategy for psoriasis. Therefore, we performed a systematic review and meta-analysis to assess the effectiveness of this combination therapy in reducing the severity of disease in patients with psoriasis.

**Methods:**

The following electronic databases were searched for articles from January 1, 2000 to March 1, 2022: PubMed, Embase, the Cochrane Central Register of Controlled Trials (CENTRAL), Chinese Biomedical Literature Database (CBM), Chinese Scientific Journal Database (VIP database), Wan-Fang Database, and China National Knowledge Infrastructure (CNKI). The language was not restricted while performing the search. The quality of articles was evaluated using Rev. Man 5.4 software (provided by the Cochrane Collaboration), comparing bloodletting cupping combined with conventional measures therapy to conventional measures treatments. The studies obtained randomized controlled trials (RCTs) of bloodletting cupping combined with conventional standard treatment for treating psoriasis. Two trained researchers (Xiaoyu Ma and Jiaming He) independently reviewed the literature, extracted data based on exclusion and inclusion criteria, and assessed the quality of the included studies. We estimated the aggregate data using a random effects model.

**Findings:**

We identified 164 studies. Ten studies met the inclusion criteria for the meta-analysis. The primary outcome indicator was the total number of effective individuals. Secondary outcomes included the Psoriasis Area and Severity Index (PASI), adverse effects, and the Dermatology Life Quality Index (DLQI). Compared with conventional treatments, bloodletting cupping combined with conventional medicine yielded an improved total effective number of persons (RR = 1.15, 95%CI: 1.07 to 1.22, *p* < 0.00001), PASI (MD = −1.11, 95%CI: −1.40 to −0.82, *p* < 0.00001) and DLQI scores (MD = −0.99, 95%CI: −1.40 to −0.59, *p* < 0.0001). We found no significant difference in adverse reactions (RR = 0.93, 95%CI: 0.46 to 1.90, *p* = 0.85). The heterogeneity test showed the total effective numbers (*p* < 0.00001, *I*^2^ = 43%) and PASI (*p* < 0.00001, *I*^2^ = 44%) and DLQI scores (*p* < 0.00001, *I*^2^ = 0%).

**Interpretation:**

Bloodletting cupping combined with conventional treatment can achieve the ideal treatment for psoriasis. However, the combined treatment in psoriasis needs to be further evaluated in high-quality RCTs with large sample sizes to enable future studies in clinical use.

## 1. Introduction

Psoriasis is a common, chronic, and inflammatory disorder characterized by a strong genetic predisposition and autoimmune pathogenic traits associated with many other medical conditions (1, 2). The worldwide prevalence varies from approximately 0.14% in East Asia to 1.99% in Oceania, with incidence and prevalence closely related to age but varying by region (3). In 2014, the WHO passed a resolution recognizing psoriasis as an incurable, chronic, non-contagious, painful, disfiguring, and debilitating disease (4). The expression of psoriasis depends on gene interaction with the environment (5). In addition, psoriasis is associated with many diseases, such as hypertension, obesity, psoriatic arthritis, depression, type 2 diabetes, and cardiovascular disease (2). In terms of medication, treatment options for psoriasis include the topical use of vitamin D analogs, glucocorticoids, keratolytics, and phototherapy. Traditional oral therapy includes cyclosporine, amitriptyline, and methotrexate. When the disease is moderate to severe, psoriasis usually requires systemic treatment (5). Patients with moderate-to-severe psoriasis have a high risk of death, mainly attributed to cardiovascular disease (6–8). Despite the availability of safe and effective treatment options for moderate-to-severe psoriasis, there is dissatisfaction with the efficacy of the treatment, underutilization, and poor adherence (9–12). A study has reported that methotrexate is associated with a high incidence rate of hepatotoxicity (13). Despite the proven efficacy of corticosteroids in treating psoriasis, studies have shown that it has potential side effects, particularly skin atrophy and adrenal suppression associated with prolonged and widespread use (14, 15). In addition, to avoid long-term immunosuppressive effects, many drugs are not allowed to be used in children, and some experts use etretinate as the treatment of choice, but long-term use can also cause skeletal changes in children (16). The presence of hepatic and renal impairment in the elderly increases the incidence of adverse reactions to cyclosporine and methotrexate (17). In addition, in patients with metabolic syndrome, drugs such as etretinate, methotrexate, and cyclosporine have been shown to have adverse effects on hypertension and liver injury (18). Biological therapies are currently emerging in the treatment of psoriasis, and Interleukin (IL)-23 inhibitors are the latest class of biological agents available for the treatment of psoriasis, which has shown good results, including showing sustainable efficacy and tolerable side effects (19, 20). Despite this, there are some safety issues or the induction of new diseases due to the diversity of patients’ conditions during the treatment (21, 22). Available Current therapies have not been shown to reverse this natural damage reliably. However, the cost is also an issue of concern. Thus, there is a pressing need for a more effective, less toxic, and cost-effective treatment to alternative therapy for psoriasis.

Bloodletting cupping, also known as blood cupping or blood-letting puncture and cupping therapy, referring to a superficial needle prick in the skin, followed by cupping, is a substantial part of complementary alternative medicine (CAM). Cupping after bloodletting can enhance the therapeutic effect of blood cupping. It treats diseases by unblocking the meridians and Qi and Blood (23). Moreover, the mechanism of cupping therapy is to influence local soft tissue microcirculation through mechanical pressure under a vacuum, which enhances capillary vascular permeability, increases regional blood circulation flow, improves metabolism, and stimulates the body’s immune response for feedback regulation (24, 25). During cupping, the most common is the appearance of cupping marks, which often appear as red petechiae or purple petechiae. Based on the above, eliminating a certain amount of blood through cupping can eliminate the accumulated harmful substances and facilitate the infusion of fresh blood. The ideal treatment would be one that can combine the ability to control the condition with a low tendency to cause adverse effects and unstable therapeutic efficacy. The further action of cupping can promote the further increase of metabolism, thus producing local and systemic regulatory results. It is mainly used to treat low back pain, soft tissue injuries or sprains, pain caused by external rheumatism, etc. Blood-letting puncture and cupping are widely used to treat psoriasis because of their relatively faster and superior effectiveness, simple manipulation, short duration of treatment, fewer adverse effects, and lower medical expenses. However, applying the method to patients with anemia, those susceptible to bleeding, or where big blood vessels lie is inadvisable (26).

Since ancient times, CAM has played an irreplaceable role in treating disease and human health and has been recognized by various countries (27). In this case, there is considerable interest in the potential benefits of bloodletting cupping combined with conventional measures therapy for psoriasis. Moreover, there is a robust clinical rationale to support such a strategy. However, its ideal role in clinical treatment strategies of effectiveness and safety on psoriasis has not been established due to the low qualities of these studies. We recognized that individual studies alone might not provide sufficient data to influence clinical practice; we attempted to assess this therapy’s potential role objectively. Therefore, we conducted a systematic review and meta-analysis of RCTs to determine the impact of combination therapy on critical outcomes such as overall effectiveness and Psoriasis Area and Severity Index (PASI) in patients with psoriasis.

## 2. Methods

We report this systematic review and meta-analysis by the PRISMA 2020 statement (28) and have registered with Prospero (number CRD42022314260).

### 2.1. Search strategy

Two researchers (Xiaoyu Ma and Minghui Zhao) independently selected comprehensive articles published between January 1, 2000 and March 31, 2022 by searching the following online databases: Embase, PubMed, the Cochrane Central Register of Controlled Trials (CENTRAL), Chinese Biomedical Literature Database (CBM), Wan-Fang Database, Chinese Scientific Journal Database (VIP database), and China National Knowledge Infrastructure (CNKI). The analysis included the total study population of the randomized, blind, and placebo-controlled trial using bloodletting cupping combined with conventional measures therapy for treating psoriasis. Two researchers (Xiaoyu Ma and Jiaming He) independently reviewed the literature against inclusion and exclusion criteria and extracted data to assess the quality of included studies. The complete detailed search string is as follows: (((“Psoriasis”[Mesh]) OR (((((Psoriasis[Title/Abstract]) OR (Psoriases[Title/Abstract])) OR (Pustulosis Palmaris et Plantaris[Title/Abstract])) OR (Palmoplantaris Pustulosis[Title/Abstract])) OR (Pustular Psoriasis of Palms[Title/Abstract] AND Soles[Title/Abstract]))) AND (((((bloodletting cupping[Title/Abstract]) OR (blood cupping[Title/Abstract])) OR (acupuncture cupping[Title/Abstract])) OR (blood-letting puncture[Title/Abstract] AND cupping[Title/Abstract])) OR (pricking[Title/Abstract] AND cupping[Title/Abstract]))) AND (((randomized controlled trial[Publication Type]) OR (randomized[Title/Abstract])) OR (placebo[Title/Abstract])).

### 2.2. Study selection and data extraction

#### 2.2.1. Study selection

We regarded studies as eligible for inclusion:

- I randomized controlled trials (RCTs)
- II at least 2 weeks duration of intervention
- III receiving bloodletting cupping combined with conventional measures therapy strategies
- IV comparing with conventional measures therapy strategies
- V outcomes including at least adverse reactions, the total effective number of people, and PASI and DLQI scores
- VI adult humans with diagnosed psoriasis of any type

The exclusion criteria were as follows:

- I involved a non-RCT design
- II participants were children
- III outcome measures were not comprehensive
- IV compared bloodletting cupping combined with conventional therapy to other treatment options

#### 2.2.2. Data extraction

First, two reviewers (Minghui Zhao and Dilong Li) independently read the title and abstract and conducted a preliminary review of the article. At the same time, a third reviewer (Jingyan Kong) decided in the event of a difference of opinion. Two researchers (Xiaoyu Ma and Jiaming He), according to the inclusion and exclusion criteria, independently examined the study by reading the full text, and a third researcher (Fang Yang) performed the assessment. Data extraction is completed by using the established extraction table. We extracted the characteristics of the following data from each eligible study: ① the first author, ② year of publication, ③ the number of cases in the treatment groups and control groups, ④ intervening measures, ⑤ treatment period (days), and ⑥ outcome indicators.

### 2.3. Assessment of risk of bias

**Figure 1:**
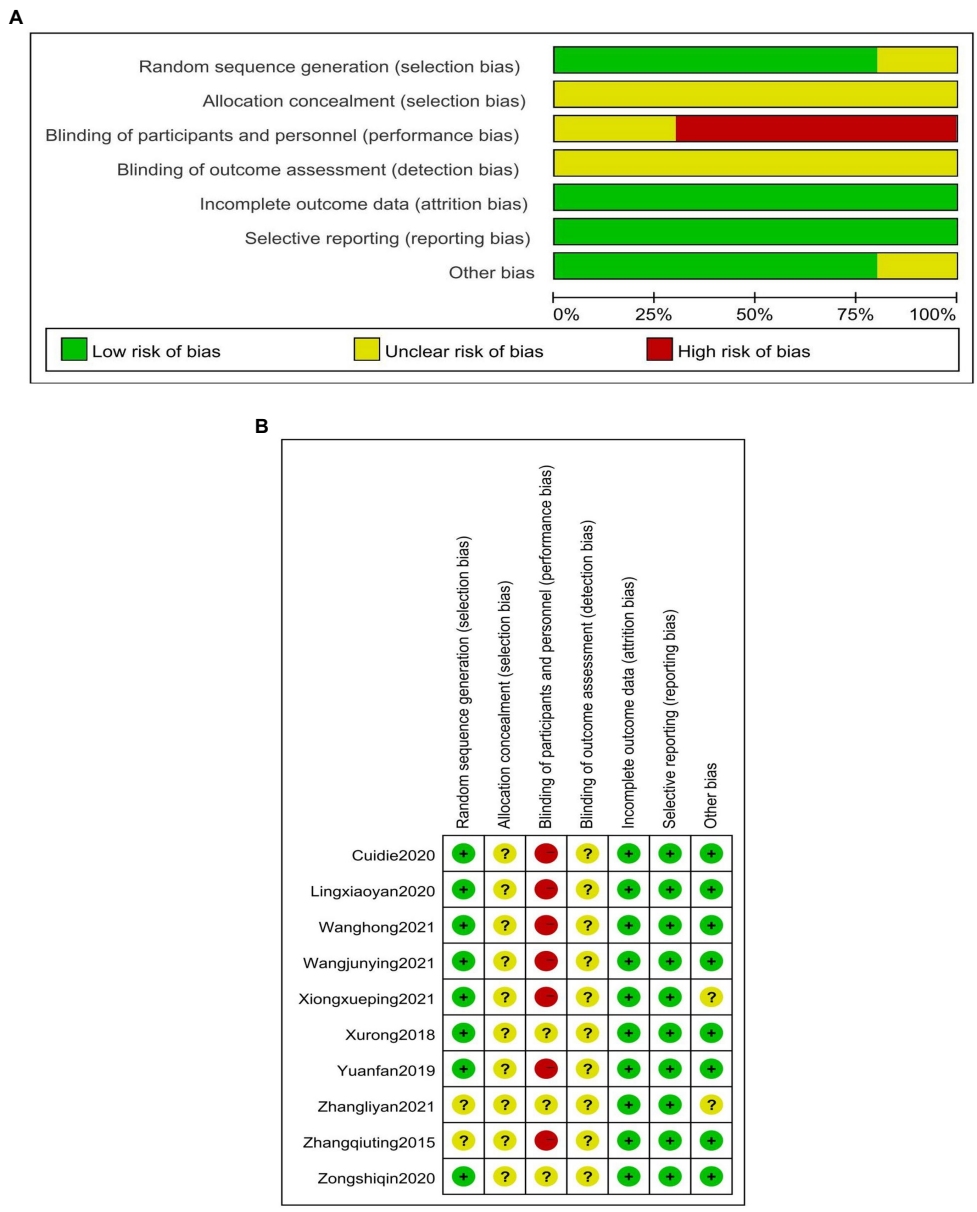
**(A)** Methodological quality assessment of the included studies. **(B)** Methodological quality assessment of the included studies.

Two researchers assessed the risk of bias according to the 7-item criteria of Rev. Man 5.4 (The Cochrane Collaboration). Two trained reviewers (Xiaoyu Ma and Dilong Li) independently assessed each included study based on its methodological quality, and disagreements were resolved through the discussion with a third author (Fan Yang). The main content of the assessment included some of the following: random allocation method, allocation options hidden, blind process, completeness of result data, selective or non-selective reporting of study results, and availability of other sources of bias (Figure 1). In the aforementioned case, a “yes” response meant a low risk of bias, a “no” response meant a high risk of bias, and an “uncertain” answer meant an unclear risk of bias (Table 1).

**Table 1:** Methodological quality evaluation of the included studies.

Studies	Random sequence generation (selection bias)	Allocation concealment (selection bias)	Blinding of participants and personnel (performance bias)	Blinding of outcome assessment (detection bias)	Incomplete outcome data (attrition bias)	Selective reporting (reporting bias)	Other bias
Cui Die, 2020 (30)	No	Unclear	Yes	Unclear	No	No	No
Ling Xiaoyan, 2020 (30)	No	Unclear	Yes	Unclear	No	No	No
Wang Hong, 2021 (30)	No	Unclear	Yes	Unclear	No	No	No
Wang Junying, 2021 (37)	No	Unclear	Yes	Unclear	No	No	No
Xiong Xueping, 2021 (32)	No	Unclear	Yes	Unclear	No	No	Unclear
Xu Rong, 2018 (34)	No	Unclear	Unclear	Unclear	No	No	No
Yuan Fan, 2019 (29)	No	Unclear	Yes	Unclear	No	No	No
Zhang Liyan, 2021 (35)	Unclear	Unclear	Unclear	Unclear	No	No	Unclear
Zhang Qiuting, 2015 (38)	Unclear	Unclear	Yes	Unclear	No	No	No
Zong Shiqin, 2020 (36)	No	Unclear	Unclear	Unclear	No	No	No

### 2.4. Statistical analysis

All RCTs were conducted with Rev. Man 5.4 software. Random effects models were used to calculate relative ratios (RR) and 95% confidence intervals (CI) for the primary outcome (dichotomous data), and mean differences (MD) and 95% CI were used to assess continuous variables. The *I*^2^ test assessed the heterogeneity of the included data if the *I*^2^ value was <50%, indicating a low statistical heterogeneity among the studies, and was accepted. Otherwise, if the *I*^2^ value was >50%, it shows a high statistical heterogeneity among the studies. The random effects model was considered for all data analysis. A funnel plot was conducted to identify the publication bias when the number of the included studies for one outcome was more than 10. We consider the primary outcome for each study was the total number of influential individuals. Secondary outcomes were adverse effects, PASI, and DLQI scores.

## 3. Results

### 3.1. Literature search results

**Figure 2:**
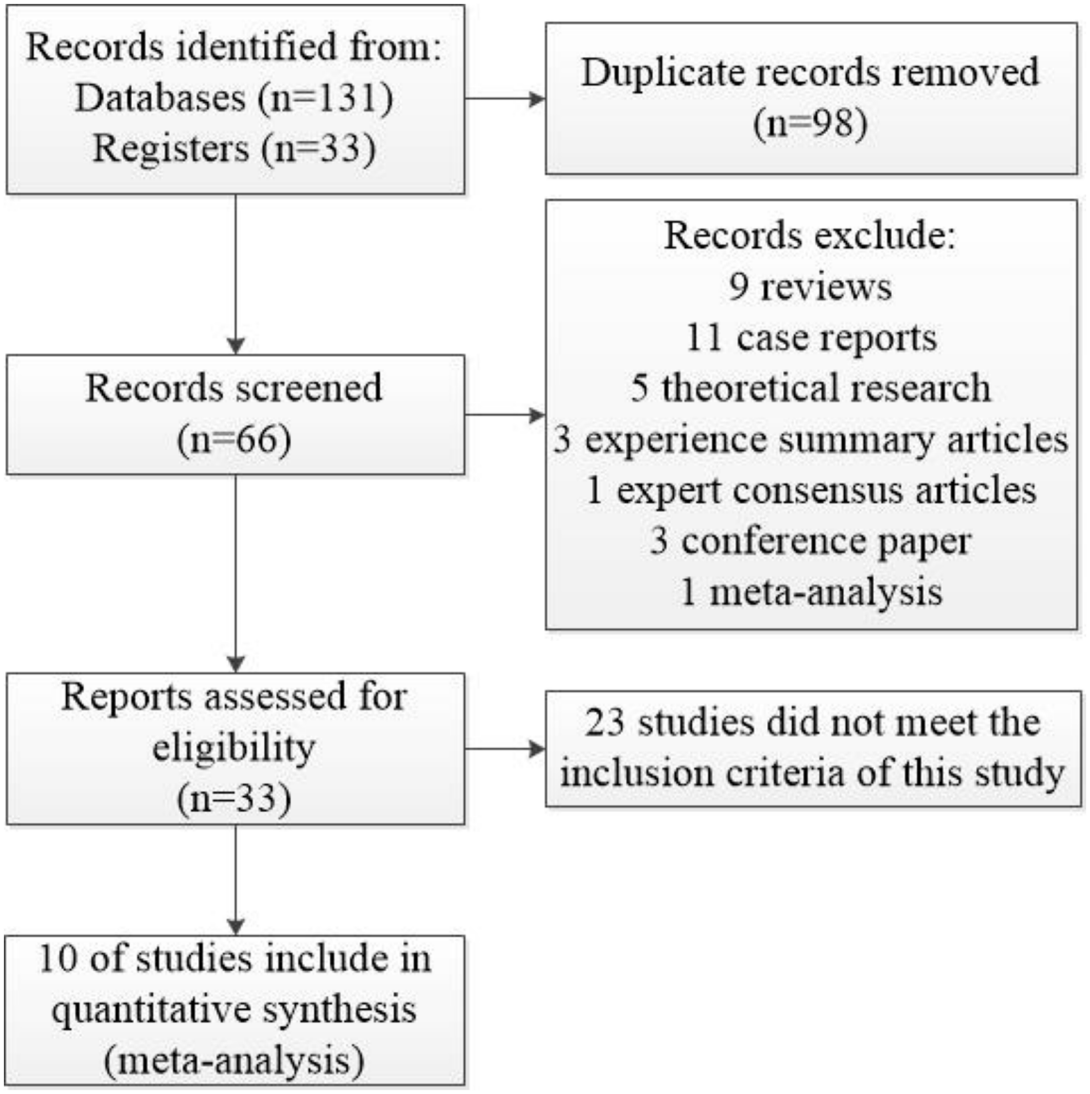
Literature selection flow chart.

We identified 164 pieces of initial literature of which 98 duplicate references were excluded and 66 were included. A total of 56 articles were excluded, screening titles and abstracts identified 33, and 23 records were excluded by reading the full text, and the screening process of the 10 included studies (29–38) is shown in Figure 2. A total of 833 patients were eligible for inclusion in the meta-analysis. Of these, 422 patients were in the treatment groups, and the other 411 patients were in other groups. In our included studies, participants in the 10 studies that met the criteria were Chinese. The study period of three studies (29–31) was 28 days, that of three studies (32–34) was 14 days, that of three studies (35, 36, 38) was 30 days, and that of only one study (37) was 90 days. We used first authors, year of publication, duration of treatment, number of cases, interventions in treatment and control groups, and outcomes as basic information for inclusion in meta-analyses. For more details, see Table 2.

**Table 2:** The basic characteristics of the included articles ① Blood-letting puncture and cupping therapy ② conventional measures therapy.

Author	Publication year	Group	Number of patients	Interventions	Treatment period (days)	Outcome indicators
Cui Die (30)	2020	Experimental group	32	① + ②	28	Total effective numbers, PASI, Traditional Chinese medicine symptom scores, DLQI Scores, Recurrence rates
		Control group	31	②	28	Number of adverse reactions
Ling Xiaoyan (30)	2020	Experimental group	45	① + ②	14	PASI, Total effective numbers, Recurrence rates
		Control group	45	②	14	
Wang Hong (30)	2021	Experimental group	38	① + ②	28	PASI, Total effective numbers, Number of adverse reactions
		Control group	38	②	28	
Wang Junying (37)	2021	Experimental group	42	① + ②	90	Total effective numbers, SDS Scores, SAS Scores, DLQI Scores, QOL Scores
		Control group	42	①	90	
Xu Rong (34)	2018	Experimental group	43	① + ②	14	PASI, Total effective numbers, Number of adverse reactions, Symptom scores
		Control group	41	②	14	
Yuan Fan (29)	2019	Experimental group	32	① + ②	28	Total effective numbers
		Control group	25	②	28	
Zhang Liyan (35)	2021	Experimental group	34	① + ②	30	Number of adverse reactions, Total effective numbers
		Control group	34	②	30	
Zhang Qiuting (38)	2015	Experimental group	38	① + ②	30	Total effective numbers
		Control group	37	②	30	
Zong Shiqin (36)	2020	Experimental group	50	① + ②	30	PASI, PQOLS scores, Number of adverse reactions, Total effective numbers
		Control group	50	②	30	
Xiong Xueping (32)	2021	Experimental group	68	① + ②	14	Total effective numbers, Patient satisfaction rates
		Control group	68	②	14	

### 3.2. Quality assessment

#### 3.2.1. Total effective numbers

**Figure 3:**
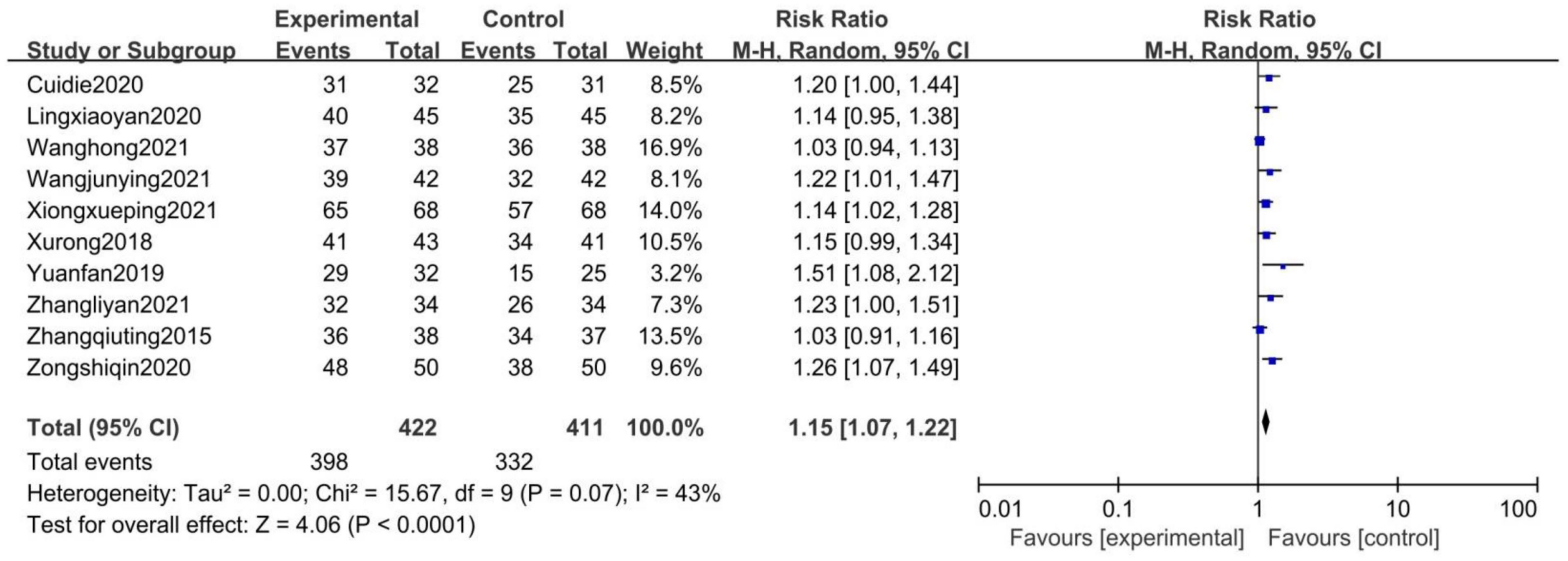
Meta analysis of total effective number between the treatment group and control group.

A total of 10 studies, with 411 participants in the control groups and 422 in the experimental groups, reported the efficacy of bloodletting puncture and cupping in combination with conventional measures for treating psoriasis. Figure 3 shows a low statistical heterogeneity (*p* < 0.00001, *I*^2^ = 43%) between the control and treatment groups. The aggregated results indicated a clear difference in the two groups (RR = 1.15, 95%CI: 1.07 to 1.22, *p* < 0.00001). Figure 3 shows the meta-analysis of efficiency between the treatment and control groups (*p* < 0.00001, *I*^2^ = 43%). Pooled results showed a significant difference between the control and treatment groups (RR = 1.15, 95% CI: 1.07 to 1.22, *p* < 0.00001). Figure 3 shows the results of the meta-analysis of the total effective numbers between the treatment and control groups.

#### 3.2.2. Psoriasis area and severity index

**Figure 4:**
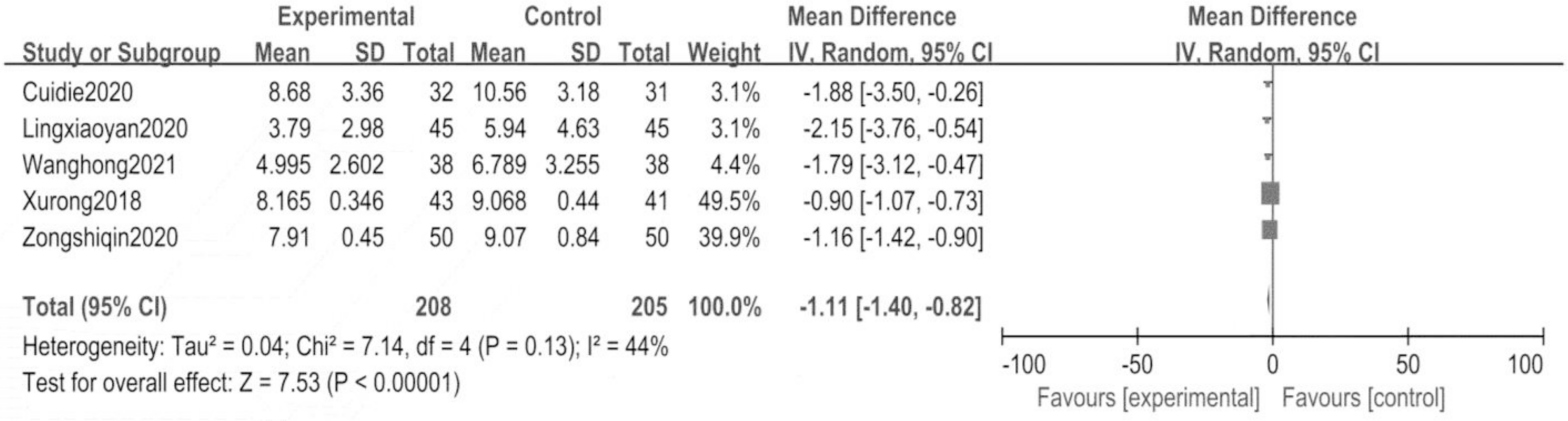
Meta analysis of PASI between the treatment group and control group.

The Psoriasis Area and Severity Index (PASI) is a combination of the severity of the lesions (including erythema, infiltrates, and scaling) and the area of the lesions for psoriasis. A specific formula is used to calculate the final score, often used to assess the severity of psoriasis, and is an internationally accepted scale for scoring the severity of psoriatic lesions (39). Of the 10 included studies, five studies involved the application of the PASI. It consisted of 208 patients in the treatment groups and 205 patients in the control groups. The *I*^2^ test was used to test for heterogeneity. We used the random effects model. The results show a low statistical heterogeneity between the two groups (*p* < 0.00001, *I*^2^ = 44%), as shown in Figure 4. The pooled results indicated a significant difference between the control and treatment groups (MD = −1.11, 95%CI: −1.40 to −0.82, *p* < 0.00001). The results are shown in Figure 4.

#### 3.2.3. Adverse reactions

**Figure 5:**
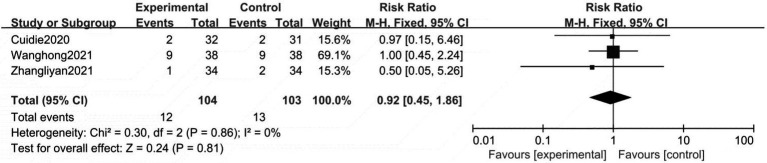
Meta analysis of number of adverse reactions between the treatment group and control group.

Only two studies reported the occurrence of adverse effects. As shown in Figure 5, no significant heterogeneity is established between the two groups (*p* = 0.85, *I*^2^ = 0%). The meta-analysis showed that the statistics were not statistically significant. Therefore, fixed effects models were used to analyze our data. The results showed a substantial difference between the control and test groups (RR = 0.92, 95% CI: 0.45 to 1.86, *p* < 0.81).

#### 3.2.4. Dermatology life quality index scores

**Figure 6:**
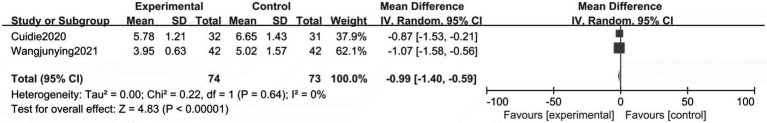
Meta analysis of DLQI score between the treatment group and control group.

For non-life-threatening psoriasis, treatment goals should focus on the patient’s perceived health-related quality of life, usually measured by the Dermatology Life Quality Index (DLQI) (40). Two studies mentioned the DLQI score, and there were 74 patients in experimental groups and 73 patients in control groups. As shown in Figure 6, significant heterogeneity is not established between the two groups (*p* < 0.00001, *I*^2^ = 0%). Meta-analysis results showed that the results were statistically significant. The combined results showed a remarkable difference between the control and test groups (MD = −0.99, 95%CI: −1.40 to −0.59, *p* < 0.00001).

### 3.3. Publication bias

**Figure 7:**
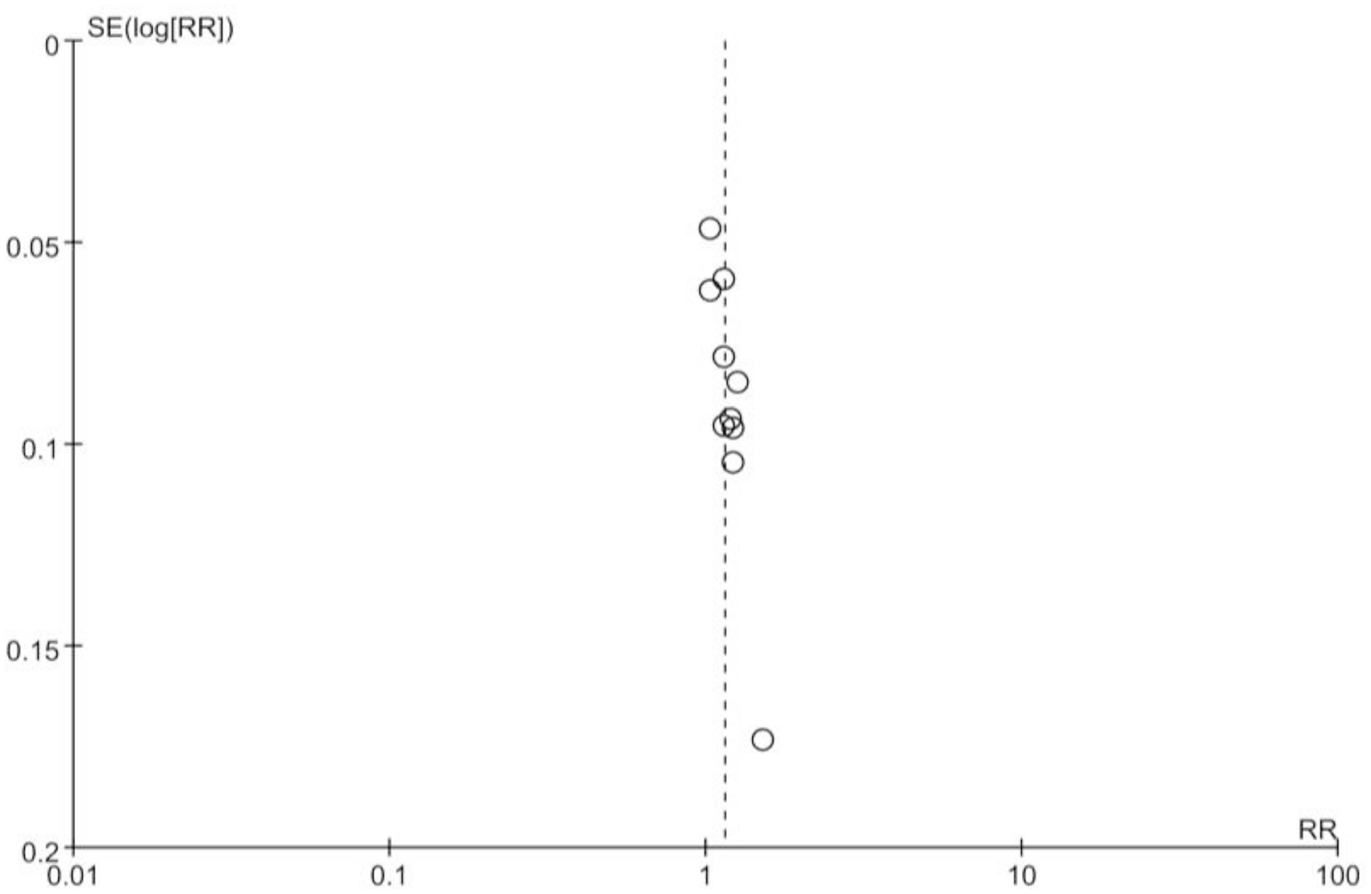
Funnel plot analysis.

The funnel plot for the total effective numbers is symmetric, indicating no significant publication bias, as presented in Figure 7.

## 4. Discussion

As far as we know, psoriasis, one of the most joint immune-mediated disorders, a papulosquamous skin disease, is distinctive, not enough to be recognized by clinicians (1). Psoriasis already possesses a substantial psychosocial barrier to patients and seriously affects their quality of life and physical and mental health (41). Frequent and long-term relapses of psoriasis have been of great concern to clinicians and healthcare professionals. The side effects of various treatments available and the unaffordable high cost of medical care mean that many patients are less satisfied with the treatment they receive. Biological therapies represent an important advance in the management of moderate-to-severe forms of plaque psoriasis, and their efficacy of them in the treatment of psoriasis has been universally recognized, specifically targeting key cytokines involved in psoriasis pathogenesis, resulting in a huge improvement of cutaneous manifestations, and with a generally safe profile. Both clinical trials and real-life studies showed impressive results for their safety and efficacy profiles. Particularly, real-life studies included patients who are typically excluded by the rigid inclusion and exclusion criteria of the clinical trials, showing significant PASI75, PASI90, and PASI 100 responses, even in more fragile patients (42–46). Despite the good performance of biological preparations, there are still some patients with poor or no efficacy. Dermatologists should identify the causes and treat them in a timely manner according to the patient’s own condition, thus making the treatment of psoriasis with biologics more professional and precise (47). Therefore, it is not surprising that in recent years, bloodletting cupping combined with conventional measures has been widely used in the treatment of psoriasis, and years of clinical experience and reports in the literature have concluded that its efficacy in psoriasis is definite, and the incidence of adverse reactions is lower compared with that of western medicine. On the one hand, trials used blood cupping therapy to unblock the meridians so that the Qi and blood flow unimpeded. On the other hand, the warm stimulation of cupping can make the pores open, and traditional Chinese medicine is called the “sweat method” so that the Qi can flow to get rid of the unhealthy trend.

In this study, a meta-analysis of the results of the combination of acupuncture cupping with other therapies compared with other therapies in the control group showed that the combination of acupuncture cupping with other therapies further reduced the PASI score and the incidence of adverse reactions compared with other control groups, and increased the total number of effective clinical patients and the DLQI score, which are indices suggesting that the medication is effective and safe.

Therefore, to provide information on the effectiveness of bloodletting cupping combined with conventional measures for psoriasis, this review was written to evaluate the currently published studies. Based on the meta-analyses, the 10 included randomized controlled trials involving 833 participants. Moreover, the results of this meta-analysis showed that, compared with conventional measures therapy alone, blood-letting puncture and cupping combined with conventional measures treatment had increased the number of clinically effective people. Meanwhile, the PASI decreased more obvious, and the Dermatology Life Quality Index (DLQI) decreased significantly. As for adverse reactions, the test groups included one case of mild diarrhea, one case of itching at the acupuncture site, nine instances of dry mouth, and one case of burning skin; the control groups had two cases of mild diarrhea, nine cases of dry mouth, and two cases of skin erythema. In addition, we found a substantial outcome difference between the control and treatment groups in terms of adverse events using a fixed effects model (*p* < 0.81), suggesting that combining the two treatments reduced the risk of adverse events. It is possible that the beneficial effects of blood-letting puncture and cupping combined with conventional measures were maybe overvalued. Most of the current clinical research literature outcome indicators are too simple and have different reference indicators, and some studies only list the total clinical effective rate, the number of adverse reactions, and PASI values. Single data cannot be meta-analyzed, so after combining all data, the total effective number, PASI, adverse reaction rate, and DLQI were finally used as valid data for the analysis.

This study has several limitations as well as relative shortcomings. First, the duration of the included trials ranged from 14 to 90 days, and no longer was efficacy observed, so it was not known whether this treatment was long-lasting. Second, the quality of the included articles was uneven. Only Chinese patients were included in the included randomized controlled trials, so there may be a potential risk of bias. Third, differences in interventions (including twice–daily versions, once–weekly versions, and twice–weekly versions) may influence the optimal choice of combination therapy. Fourth, the grey literature did not search, and publication bias may exist. Fifth, the differences in blooding and cupping techniques used by doctors, such as the amount of blood released, the strength of the cupping, and the depth of the needles, can also impact the efficacy to some extent. In addition, patient satisfaction, recurrence rates, and other issues related to the bloodletting puncture and cupping with conventional treatment measures have been up in the air. Finally, it is unknown when in the course of clinical treatment is the best time to start this treatment.

Although more robust evidence is needed to determine the best way and method to apply this integrative treatment approach in clinical practice, our findings support the use of bloodletting cupping combined with conventional measures therapy during the clinical trial in patients with psoriasis.

## Data availability statement

The original contributions presented in the study are included in the article/supplementary material, further inquiries can be directed to the corresponding authors.

## Author contributions

FY and XM proposed and designed this study. XM, MZ, and DL retrieved and selected the data. XM and JH were responsible for the extraction of data and the quality assessment of all study data. XM then performed a statistical analysis and summarized. XM drafted the manuscript and then FY and JK revised it. All authors contributed to the article and approved the submitted version.

## Conflict of interest

The authors declare that the research was conducted in the absence of any commercial or financial relationships that could be construed as a potential conflict of interest.

## Publisher’s note

All claims expressed in this article are solely those of the authors and do not necessarily represent those of their affiliated organizations, or those of the publisher, the editors and the reviewers. Any product that may be evaluated in this article, or claim that may be made by its manufacturer, is not guaranteed or endorsed by the publisher.
